# Ethnic differences in quality of life and its association with survival in patients with heart failure

**DOI:** 10.1002/clc.23394

**Published:** 2020-06-20

**Authors:** Gillian Stein, Tiew‐Hwa K. Teng, Wan T. Tay, A. Mark Richards, Robert Doughty, YanHong Dong, David Sim, Poh S. D. Yeo, Fazlur Jaufeerally, Gerard Leong, Dinna Soon, Lieng H. Ling, Carolyn S. P. Lam

**Affiliations:** ^1^ NYU Grossman School of Medicine New York New York USA; ^2^ National Heart Centre Singapore Singapore Singapore; ^3^ Duke‐NUS Medical School Singapore Singapore; ^4^ School of Population & Global Health University of Western Australia Perth Western Australia Australia; ^5^ National University Heart Centre Singapore Singapore; ^6^ University of Auckland Auckland New Zealand; ^7^ National University Health System Singapore Singapore; ^8^ Singapore General Hospital Singapore Singapore; ^9^ Gleneagles Medical Centre Singapore Singapore; ^10^ Mount Elizabeth Hospital Singapore Singapore; ^11^ Khoo Teck Puat Hospital Singapore Singapore; ^12^ Department of Cardiology University Medical Center Groningen Groningen The Netherlands

**Keywords:** ethnicity, heart failure, quality of life, survival

## Abstract

**Background:**

Optimizing quality of life (QoL) is a key priority in the management of heart failure (HF).

**Hypothesis:**

To investigate ethnic differences in QoL and its association with 1‐year survival among patients with HF.

**Methods:**

A prospective nationwide cohort (n = 1070, mean age: 62 years, 24.5% women) of Chinese (62.3%), Malay (26.7%) and Indian (10.9%) ethnicities from Singapore, QoL was assessed using the Minnesota Living with HF Questionnaire (MLHFQ) at baseline and 6 months. Patients were followed for all‐cause mortality.

**Results:**

At baseline, Chinese had a lower (better) mean MLHFQ total score (29.1 ± 21.6) vs Malays (38.5 ± 23.9) and Indians (41.7 ± 24.5); *P* < .001. NYHA class was the strongest independent predictor of MLHFQ scores (12.7 increment for class III/IV vs I/II; *P* < .001). After multivariable adjustment (including NT‐proBNP levels, medications), ethnicity remained an independent predictor of QoL (*P* < .001). Crude 1‐year mortality in the overall cohort was 16.5%. A 10‐point increase of the physical component (of MLHFQ) was associated with a hazard (HR 1.22, 95% 1.03‐1.43) of 1‐year mortality (*P* = .018) in the overall cohort. An interaction between MLHFQ and ethnicity was found (*P* = .019), where poor MLHFQ score (per 10‐point increase) predicted higher adjusted mortality only in Chinese (total score: HR 1.18 [95% CI 1.07‐1.30]; physical: HR 1.44 [95% CI 1.17‐1.75]; emotional score: HR 1.45 [95% CI 1.05‐2.00]).

**Conclusions:**

Ethnicity is an independent determinant of QoL in HF. Despite better baseline QoL in Chinese, QoL was more strongly related to survival in Chinese vs Malays and Indians. These findings have implications for HF trials that use patient‐reported outcomes as endpoints.

## 1 INTRODUCTION

Heart failure (HF) is a debilitating condition and a leading cause of mortality worldwide.^1^ Increasingly, symptomatic HF appears to affect patients in Southeast Asia disproportionately, with HF presenting at a much younger age, characterized by greater severity and poorer outcomes when compared to the rest of the world.^2^ Within the Southeast Asian patient population with HF, there exist drastic ethnic differences in both hospitalization rates and mortality.^2^ Understanding these differences and their underlying mechanisms—social and pathophysiologic—is necessary to effectively treat the growing HF disease burden in these countries.

Multi‐morbidity is a hallmark of HF patients; their coexistence and interactions impact outcomes and functional status adversely in the elderly.^3^ To effectively address HF, it is therefore imperative to gain a deeper understanding of how it affects patients' quality of life (QoL), as a cross‐disease outcome.^4^, ^5^ For this study we focused on patient‐reported QoL, one aspect of patient reported outcome measures (PROMs). This choice builds on a growing recognition that appropriate decisions can only be made when informed by both biomedical factors *and* patient concerns.^6^ The increasing importance of patient input has largely been fueled by the growing burden of chronic disease, for which care is often long‐term and costly. While evidence remains inconsistent, some studies have found that the use of PROMs positively impacted health outcomes.^6^ The integration of PROMs into clinical care represents a unique opportunity to improve the patient experience, the doctor‐patient relationship, and ultimately, health outcomes.

HF has a notoriously negative impact on patient‐reported QoL.^7^, ^8^, ^9^, ^10^ Patients must cope not only with their physical symptoms, including shortness of breath and fatigue, but also with the inability to do the things they once could, the emotional stress of being sick, and the financial burden of treatment. Accordingly, several HF‐specific QoL measurement tools have been developed over the years.^11^, ^12^, ^13^, ^14^, ^15^ Studies show that a majority of HF patients place greater value in QoL than longevity.^16^ Optimizing QoL, as a patient‐centered outcome, must therefore become a key priority in the management of HF. In addition to being a significant treatment aim, QoL also has the potential to be a useful prognostic tool for HF, although past studies have reported inconsistent findings on the association between poor QoL and worse survival.^17^ Despite a wealth of data on QoL for Western patients with HF, where prevalence is ~1% to 2%, there is a notable lack of data for Southeast Asian patients with HF.^2^, ^18^

We sought to investigate ethnic differences in QoL by looking at QoL through three distinct lenses: descriptive, causal, and prognostic. We investigated potential interethnic differences in QoL and assessed the relationship of QoL to mortality.

## 2 METHODS

### 2.1 Setting

Singapore is a highly developed city state. Its national healthcare expenditure constitutes approximately 4.9% of the GDP, which is considerably lower than other developed countries.^19^ Singapore extends universal healthcare coverage to all the citizens; however, it has a mixed healthcare financing system.^20^ While public expenditure on healthcare is partially financed through general revenues, the healthcare financing system has been layered with a more elaborate diversified system through legislating compulsory savings funds by private individuals to fund healthcare expenditure.^20^ Consequently, out‐of‐pocket healthcare expense in Singapore is relatively high (54.8%) compared with other developed countries.^19^ Nonetheless, multiple layers of healthcare financing and government subsidies (up to 80% of total bill) are in place to ensure that local citizens are not denied access to healthcare.^21^

### 2.2 Study design and study population

Data from the Singapore Heart Failure Outcomes and Phenotypes study (SHOP) study^18^ were used to address the aims of this study. The SHOP study was a population‐based study of patients with a validated diagnosis (clinician‐judged) of HF, who were recruited from six centers in Singapore. Patients above 18 years of age who either presented to the hospital with a principal diagnosis of HF or attended an outpatient hospital clinic for the management of HF—within 6 months from an episode of acute decompensated HF that resulted in a hospital admission or was treated in the outpatient clinic—were included into the study and followed prospectively for 1‐year. Patients with severe valvular heart disease, transient pulmonary oedema complicating acute coronary syndrome, or end‐stage renal disease were excluded from the study.

The demographics of Singapore and HF admissions had been previously described.^2^, ^18^ The major ethnic groups comprised: Chinese (74%), Malay (13%), and Indian (9%), respectively, of the 5.5 million population.^18^

Baseline patient demographic details and clinical data, such as vital signs and symptoms on physical examination, New York Heart Association (NYHA) functional status, serum biochemistry and hematology, comorbid conditions, medications, and interventions were recorded. Comprehensive two‐dimensional echocardiography was also performed on all eligible patients, using standardized machines at study sites.

Health‐related QoL was assessed at baseline, 3 and 6 months following first admission/consultation with the Minnesota Living with Heart Failure Questionnaire (MLHFQ).^11^ The MLHFQ^11^ is a self‐report questionnaire comprising 21 items, which assesses how HF affects the physical and emotional dimensions of the well‐being of the patient. These dimensions are combined into a total score that reflects a global assessment of the patient's well‐being. Patients were asked to indicate how much HF prevented them from living, using an ordinal scale from 0 (not present or no effect), 1 (very little), up to 5 (very much). The MLHFQ score, computed by the summation of the scores to all the questions, ranges from a minimum of zero which equates to no impairment as a consequence of HF and 105 for maximum impairment. Lower MLHFQ scores correlate with better QoL. The questions cover signs and symptoms pertaining to physical activity, social interaction, sexual activity, work, and emotions. The MLHFQ was administered by the team of multi‐ethnic clinical coordinators who translated it into Mandarin, Malay, Tamil (as the official language for South Asian language in Singapore) and other common dialects, for non‐English speaking patients. The validity of the MLHFQ has been well documented.^22^

For socioeconomic status, we used the small areal Socioeconomic disadvantage index (SEDI) described by Earnest et al.^23^ Data sources used for the derivation of SEDI were obtained from the 2010 Singapore Census of Population, and the Singapore Master Plan geographical boundary level, which is used by the Urban Redevelopment Plan (URA) authorities for town planning purposes. Using 23 variables based on a person's place of residence indicative of socioeconomic status (housing type, highest educational level, literacy level, occupational categories, industries employed in, and personal and household income), Earnest et al derived SEDI by principal component analysis in a structured and iterative process. In the current study, SEDI values could then be assigned to each participant based on his/her Singapore residential address (indicated by postal codes).

## 3 STATISTICAL ANALYSIS

Descriptive statistics were used to characterize the study population and ethnic groups. Categorical variables are described as percentages and continuous variables are described as a mean with standard deviations or median (interquartile range) if skewed. The relationship of MLHFQ scores (total, physical, and emotional) with independent risk factors was assessed using linear least‐squares regression models. Univariable analyses were first performed on all the baseline variables. Covariates with *P*‐values <.1 were then considered for multivariable adjustments, including any important clinical and demographic factors.

Multivariable Cox proportional hazards models were used to determine the association of MLHFQ scores (total, physical, and emotional) with 1‐year all‐cause mortality. Multivariable adjustments included ethnicity, age, sex, body mass index, systolic blood pressure, diastolic blood pressure, heart rate, biomarkers (Galectin‐3, NT‐proBNP), NYHA functional class, diabetes, coronary artery disease, atrial fibrillation, hypertension, prior stroke, liver disease, chronic respiratory disease, history of smoking or alcohol usage, evidence‐based medications for HF, and areal socio‐economic disadvantage index (SEDI),^23^ as proxy for socio‐economic status. Interaction effects were checked. A two‐tailed *P* value of less than .05 was considered statistically significant. All statistical analyses were performed with STATA/SE v14.0.

Ethics approvals were obtained from the relevant human ethics committees at the investigating sites. The study conforms to the Declaration of Helsinki.

## 4 RESULTS

### 4.1 Subject characteristics

Baseline characteristics of 1070 HF patients (age 62.0 ± 12.1, 24.5% women, mean LVEF 34.8%, 22% HF with preserved ejection fraction) are summarized in Table 1. Of the patients studied, 62.3% were Chinese, 26.7% were Malay, and 10.9% were Indian. Chinese patients were older than Malay and Indian patients (63.4 ± 12.5, vs 59.3 ± 10.8 and 60.2 ± 11.0, *P* < .001), with the proportions of ≥65 years being 46.1% in Chinese, 30.5% in Malays, and 32.3% in Indians, respectively. No significant difference (*P* = .149) in areal socio‐economic disadvantage index (as proxy for socio‐economic status) was observed among the three ethnicities, with 72.5% Chinese, 76.7% Malay, and 68.3% Indian, being in the lowest two SEDI quartiles.

**TABLE 1:** Baseline characteristics, by ethnic groups

	All	Chinese	Malay	Indian	*P* value
n	1070	667	286	117	
Age, years	62.0 ± 12.1	63.4 ± 12.5	59.3 ± 10.8	60.2 ± 11.0	<.001
Sex, female %	24.5	23.8	25.2	26.5	.786
Clinical characteristics					
Body mass index, kg/m^2^	26.3 ± 5.5	25.5 ± 5.0	27.8 ± 6.1	27.2 ± 5.7	<.001
Systolic blood pressure, mm Hg	125.2 ± 22.4	125.3 ± 22.5	125.8 ± 22.6	122.8 ± 20.7	.456
Diastolic blood pressure, mm Hg	71.4 ± 12.9	71.3 ± 13.0	71.7 ± 12.5	71.2 ± 13.1	.869
Heart rate, bpm	76.3 ± 13.9	76.1 ± 14.0	75.9 ± 13.4	78.5 ± 14.2	.179
eGFR, mL/min/1.73 m^2^	61.2 ± 25.9	60.4 ± 25.3	61.2 ± 27.1	65.7 ± 25.9	.149
Biomarkers					
Galectin‐3	7.8 ± 2.5	7.8 ± 2.4	7.9 ± 2.6	7.3 ± 2.8	.104
NT‐proBNP	4045.2 ± 5730.9	4164.9 ± 5560.0	3917.4 ± 5893.0	3651.1 ± 6326.8	.630
Comorbidities					
NYHA					.062
Class I	25.0	27.5	20.2	21.7	
Class II	58.6	57.7	62.8	53.9	
Class III	14.9	13.4	15.2	22.6	
Class IV	1.5	1.4	1.8	1.7	
Coronary artery disease, yes %	53.8	49.2	59.4	66.7	<.001
Atrial fibrillation, yes %	23.1	26.5	19.5	12.2	.001
Hypertension, yes %	72.2	69.4	77.4	75.9	.027
Prior stroke, yes %	11.0	10.3	10.2	16.2	.154
Peripheral arterial vascular disease, yes %	4.9	3.8	6.4	7.7	.076
Chronic respiratory disease, yes %	8.5	7.2	10.2	12.0	.115
Diabetes, yes %	57.1	50.7	65.1	74.4	<.001
Liver disease, yes %	4.1	4.7	3.2	3.4	.526
Cancer, yes %	3.3	3.7	3.6	0.9	.296
Smoking, yes %	53.8	52.4	59.7	47.4	.040
Alcohol, yes %	32.1	36.8	18.0	39.7	<.001
Medical therapy					
ACE inhibitor, yes %	58.8	57.6	63.3	54.7	.165
Angiotensin II receptor blocker, yes %	12.1	11.2	11.9	17.9	.121
β‐blockers, yes %	87.5	88.2	88.8	80.3	.045
Diuretics, yes %	89.6	89.2	90.2	90.6	.839
Digoxin, yes %	25.2	26.7	20.3	29.1	.068
Statin, yes %	83.4	80.8	89.9	82.1	.002
LVEF, %	34.7 ± 15.4	34.6 ± 15.7	34.8 ± 14.9	34.6 ± 14.9	.559
Socioeconomic status, %					
Low income	24.2	24.5	24.3	21.4	.149
Lower‐middle income	49.0	48.0	52.5	46.6	
Middle‐upper income	21.9	21.2	21.2	28.1	
High income	4.9	6.2	2.0	3.9	
Minnesota Living with Heart Failure					
Total score	32.9 ± 23.1	29.1 ± 21.6	38.5 ± 23.9	41.7 ± 24.5	<.001
Physical component score	15.4 ± 11.0	13.9 ± 10.5	17.3 ± 11.4	19.5 ± 11.2	<.001
Emotional component score	5.9 ± 6.0	5.1 ± 5.6	7.0 ± 6.1	7.5 ± 6.4	<.001

Comorbidities were common in the study population (Table 1): with high prevalence of coronary artery disease (CAD) (in 53.8%), hypertension (72.2%), diabetes (57.1%), chronic kidney disease (CKD, 50.5%) and smoking (53.8%). CAD, hypertension, and diabetes were significantly higher in Malays and Indians vs Chinese (*P* < .001). Comorbid atrial fibrillation was highest in the Chinese (26.5%), but notably the least in Indians (12.2%). CKD was more similarly prevalent in half of Chinese and Malays but less in Indians (44.6%). For lifestyle risk factors, Malays were more likely to be smokers than Chinese and Indians, but Indians were more likely to report alcohol intake than the other ethnicities (*P* < .001), Table 1.

Chinese also had lower BMIs than Malay and Indian patients (*P* < .001). Systolic and diastolic blood pressure, heart rate, eGFR, galectin‐3 levels, NT‐proBNP levels, sex, and NYHA class composition did not differ significantly across ethnicities, although more Malay (17.0%) and Indian (24.3%) patients had higher severity of HF (in NYHA class III/IV) compared to Chinese (14.8%).

A majority of patients took ACE inhibitors or ARBs (70.1%), β‐blockers (87.5%), diuretics (89.6%), and statins (83.4%). Malay patients were more likely to be on statins than Chinese and Indian patients (89.9% vs 80.8% and 82.1%, *P* = .002). Indian patients were less likely than Chinese or Malay patients (80.3% vs 88%‐89%) to be on β‐blockers, despite higher prevalence of CAD or hypertension than Chinese or Malay patients (*P* = .045).

### 4.2 Quality of life

At baseline, mean MLHFQ scores in the entire cohort for the total, physical, and emotional components were 32.9 ± 23.1, 15.4 ± 11.0, and 5.9 ± 6.0, respectively. Chinese had a lower (better) MLHFQ total score (29.1 ± 21.6) compared with Malays (38.5 ± 23.9) and Indians (41.7 ± 24.5); *P* < .001 (Table 1). Chinese patients also had lower MLHFQ physical and emotional scores compared to Malays and Indians (*P* < .001; Table 1). After adjusting for NYHA class, demographics, NT‐proBNP levels, comorbidities and medications, ethnicity remained a strong independent predictor of QoL (*P* < .001). Left ventricular ejection fraction was not independently associated with QoL.

### 4.3 Responses to MLHFQ

Of the 21 MLHFQ questions, patients reported the greatest burden in response to “Costing you money for medical care?” (median: 3, Table 2). Patients also reported a substantial burden in response to questions focused strictly on physical symptoms: “making your walking about or climbing stairs difficult?”, “making you short of breath?”, and “making you feel tired, fatigued or low on energy?” (median: 2). The lowest scores were given in response to “making it difficult for you to concentrate or remember things?”, “making you feel depressed?” and “making your sexual activities difficult” (median: 0).

**TABLE 2:** Minnesota living with heart failure questionnaire—Item response by ethnicity

Did your heart failure prevent you from living as you wanted during the last month by:		No. of valid responses	Mean responses				Median responses			
			All	Chinese	Malay	Indian	All	Chinese	Malay	Indian
1	Causing swelling in your ankles, legs, and so on?	1069	1.6	1.5	1.7	2.1	1	1	1	2
2	Making you sit or lie down to rest during the day?	1068	1.9	1.7	2.1	2.2	2	1	2	2
3	Making your walking about or climbing stairs difficult?	1069	2.4	2.2	2.6	2.9	2	2	3	3
4	Making your working around the house or yard difficult?	1070	1.6	1.4	1.8	2.1	1	1	2	2
5	Making your going places away from home difficult?	1070	1.9	1.7	2.2	2.5	2	1	2	3
6	Making your sleeping well at night difficult?	1070	1.9	1.8	2.2	2.3	2	1	2	2
7	Making your relating to or doing things with your friends or family difficult?	1070	1.4	1.2	1.6	1.9	1	0	1	2
8	Making your working to earn a living difficult?	1068	1.3	1.1	1.7	1.8	0	0	1	1
9	Making your recreational pastimes, sports or hobbies difficult?	1069	1.2	1.0	1.7	1.5	0	0	1	0
10	Making your sexual activities difficult?	1048	1.0	0.7	1.5	1.3	0	0	1	0
11	Making you eat less of the foods that you like?	1067	1.4	1.2	1.8	2.0	1	0	2	2
12	Making you short of breath?	1070	2.2	2.0	2.4	2.7	2	2	3	3
13	Making you feel tired, fatigued or low on energy?	1070	2.1	1.9	2.4	2.8	2	2	3	3
14	Making you stay in hospital?	1067	1.9	1.7	2.1	2.3	2	1	2	3
15	Costing you money for medical care?	1070	2.4	2.2	2.8	2.7	3	2	3	3
16	Giving you side effects from medications?	1069	1.0	0.8	1.2	1.3	0	0	0	1
17	Making you feel you are a burden to your family or friends?	1069	1.3	1.1	1.6	1.5	0	0	1	1
18	Making you feel a loss of self‐control in your life?	1069	1.2	1.1	1.4	1.7	1	0	1	1
19	Making you worry?	1070	1.8	1.6	2.0	2.2	1	1	2	2
20	Making it difficult for you to concentrate or remember things?	1070	0.8	0.7	0.9	0.9	0	0	0	0
21	Making you feel depressed?	1070	0.9	0.7	1.1	1.2	0	0	1	0

Among patients in the two lowest SEDI quartiles, 52% reported substantial economic burden of HF (with scores ≥3), with significantly more Malays (41.2%) and Indians (39.3%) than Chinese (29.3%), *P* < .001, expressing substantial economic burden (scores ≥4) in response to “Costing you money for medical care?”.

### 4.4 Factors associated with MLHFQ scores

Table 3 shows the relationship of MLFHQ scores and independent factors, after multivariable adjustment for the listed variables. Being female was associated with higher (worse) MLHFQ total, physical, and emotional scores. After multivariable adjustment for demographics, clinical characteristics, log NT‐proBNP levels, comorbidities, and medications, NYHA class III/IV (vs Class I/II) was the single most powerful independent predictor of MLHFQ scores: total (*β* = 12.7 units increment), physical (6.2 units), and emotional (2.3 units), respectively. Ethnicity, higher log NT‐proBNP levels, and comorbidities (eg, liver disease, peripheral arterial disease, diabetes) were also independently associated with increased MLHFQ scores. Evidence‐based medications and statins were negatively associated with MLHFQ (Table 3), consistent with improvement in QoL.

**TABLE 3:** Relationship of MLHFQ scores and independent factors

	Total score			Physical score			Emotional score		
	Beta	SE	*P*‐value	Beta	SE	*P*‐value	Beta	SE	*P*‐value
Ethnicity, vs Chinese									
Malays	8.12	1.64	<.001	3.41	0.79	<.001	1.54	0.44	.001
Indians	12.22	2.30	<.001	5.26	1.11	<.001	2.10	0.63	.001
Age, years	−0.09	0.07	.169	0.03	0.03	.371	−0.04	0.02	.013
Sex, female vs male	3.97	1.64	.016	2.82	0.81	.001	0.98	0.44	.027
Clinical characteristics									
Body mass index, kg/m^2^	0.51	0.14	<.001	0.27	0.07	<.001	0.08	0.04	.030
Systolic blood pressure, mm Hg	−0.10	0.03	.002	−0.05	0.01	.002	−0.02	0.01	.046
Heart rate, bpm	0.03	0.05	.532	0.01	0.02	.709	0.00	0.01	.781
eGFR, mL/min/1.73 m^2^	0.04	0.03	.154	0.02	0.01	.162	0.00	0.01	.921
Biomarkers									
Log NT‐proBNP	4.20	0.55	<.001	2.05	0.27	<.001	0.70	0.15	<.001
Comorbidities									
NYHA, class III/IV vs class I/II	12.66	1.88	<.001	6.21	0.90	<.001	2.30	0.51	<.001
Atrial fibrillation, yes vs no				0.27	0.82	.741			
Prior stroke, yes vs no							1.40	0.59	.018
Peripheral arterial vascular disease, yes vs no	4.80	3.22	.136	2.29	1.55	.141	0.54	0.87	.536
Diabetes, yes vs no	1.76	1.42	.217	0.68	0.68	.316	0.62	0.38	.105
Liver disease, yes vs no	8.39	3.49	.016	3.87	1.65	.019	1.51	0.94	.109
Alcohol, yes vs no				1.69	0.76	.026			
Medical therapy									
ACE inhibitor, yes vs no	−1.89	1.55	.224	−0.89	0.74	.229	−0.01	0.42	.983
Angiotensin II receptor blocker, yes vs no	−3.88	2.31	.093	−2.14	1.09	.049	0.09	0.62	.883
β‐blockers, yes vs no	−2.14	2.14	.317	−0.82	1.02	.421	−0.45	0.58	.434
Diuretics, yes vs no	3.23	2.32	.165	1.59	1.10	.147			
Digoxin, yes vs no							0.76	0.43	.078
Statin, yes vs no	−3.97	1.91	.038	−1.91	0.90	.034	−0.90	0.52	.083
Middle‐upper and high vs low‐lower middle income category	1.61	1.59	.313	1.17	0.74	.115	0.06	0.44	.888

*Note:* Adjusted for listed variables for the respective scores.

### 4.5 MLHFQ scores at 6 months' follow‐up

MLHFQ scores for the total, physical, and emotional components at 6 months were available for 622, 647, and 645 patients, respectively. At 6 months' follow‐up, all ethnic groups showed improvement in QoL, with a cohort‐wide total score change of −11.0 ± 22.5 (Tables S1 and S2). The greatest improvement was seen in Indians (−18.3 ± 21.3) as compared to Malays (−11.1 ± 25.3) and Chinese (−9.9 ± 21.3). At 6 months, Indians improved such that the average MLHFQ total score was not significantly different from the Chinese (*P* = .32).

### 4.6 One‐year mortality

**FIGURE 1:**
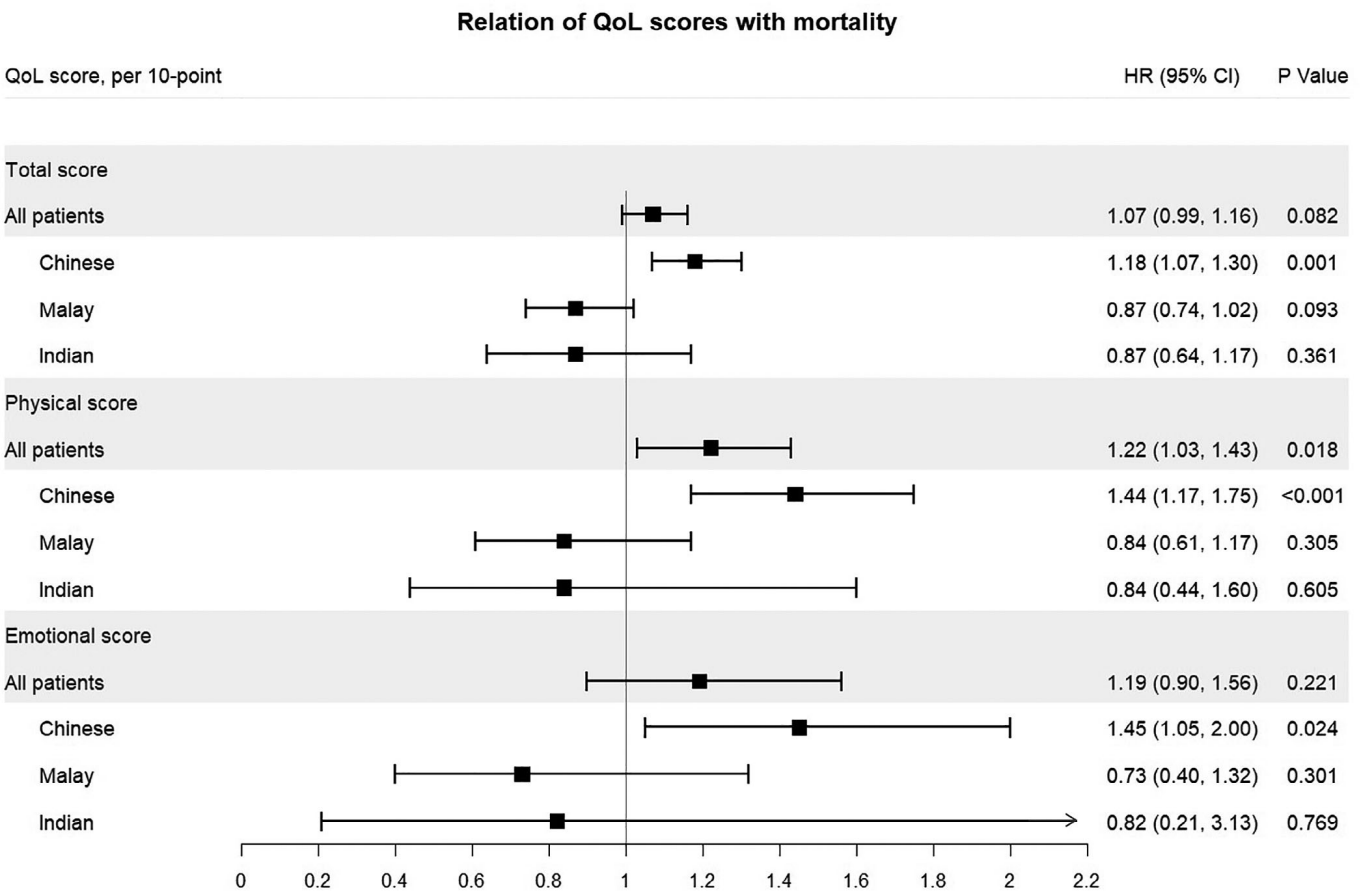
Association of QoL with 1‐year all‐cause mortality. Adjusted for age, sex, race, BMI, systolic blood pressure, heart rate, eGFR, log NT‐proBNP, diabetes, ACE inhibitors/ARB, β‐blockers and statins

Crude 1‐year all‐cause mortality was 16%, with no significant difference among the ethnicities. Of the three scores: MLHFQ total, physical, and emotional scores, the physical score was the strongest independent predictor of mortality (Figure 1). The highest (vs first) quartile of physical MHLFQ score was significantly associated with an increased hazard of adjusted 1‐year mortality (HR = 2.08 [95% CI 1.17‐3.70]; *P* = .013). Furthermore, a significant interaction between the MLHFQ total score and ethnicity was found (*P* = .019), where a poor MLHFQ score predicted higher adjusted mortality (per 10‐unit increment) only in Chinese (HR = 1.18 [95% CI 1.07‐1.30]) for total score, HR =1.44 (95% CI 1.17‐1.75) for physical and HR = 1.45 (95% CI 1.05‐2.00) for emotional scores, respectively.

## 5 DISCUSSION

This prospective, population‐based study examines multi‐ethnic differences in the perception and impact of HF through the lens of patient‐reported QoL. The principal findings of this study indicate that among this relatively young and diverse HF cohort of Asian patients with a heavy burden of comorbidities, ethnicity, independent of NYHA class and NT‐proBNP values, is an independent determinant of QoL. There was a significant, interethnic difference in patients' experience of HF, where Chinese patients consistently reported better total, physical, and emotional QoL than Indian and Malay patients. Physical (domain) QoL score had the strongest association with 1‐year adjusted mortality than total and emotional QoL scores. Significant interactions between MLHFQ total and physical scores with ethnicity on 1‐year adjusted mortality were however observed such that Chinese had poorer outcomes than Malays or Indians, independent of NT‐proBNP and contemporary evidence‐based pharmacotherapy for HF. Interestingly, all three ethnic groups reported significant improvement (greatest in Indian patients) in overall QoL, with no significant ethnic difference in QoL at 6 months. This finding suggests that the receipt of medical care ameliorated ethnic differences in QoL for our study subjects.

There has been a growing recognition that PROMs are a legitimate measure for monitoring health care outcomes. PROMs can provide insights that, while unobtainable through direct clinical measurements, are nonetheless consistent with those of clinicians.^24^ Notably, NHYA class III/IV (vs class I/II) was observed to be the single most powerful independent predictor of MLHFQ total score, suggesting that the findings confirmed a certain level of alignment between physician reported and patient reported QoL, which had been previously documented.^24^

Physical mobility contributed to a significant component of the overall MLHFQ score. In terms of its prognostic utility, we observed a strong (twofold) association of a high (fourth quartile vs first quartile) physical score with 1‐year adjusted mortality in the overall cohort. Findings from several studies with inclusion of the physical domain of the MLHFQ had been inconsistent in terms of its predictive association with mortality.^25^, ^26^, ^27^, ^28^, ^29^ Our findings were consistent with three of these studies^25^, ^28^, ^29^ which had similarly found physical mobility to be independently associated with mortality. Other remaining studies^26^, ^27^ where no association was found had relatively smaller sample sizes.

Intriguingly, a significant interaction between the overall MLHFQ score and ethnicity with respect to mortality was observed where poor MLHFQ score predicted higher adjusted mortality *only* in the Chinese patients (compared to Malays and Indians). The significantly older age of the Chinese (63.4 years) vs other ethnicities (59.3 years in Malays; 60.2 years in Indians), higher comorbidity burden (particularly CKD), and the lower uptake of ACEi/ARBs could in part explain the variation. Additionally, there might be residual confounding of unmeasured clinical factors with the outcome examined.

To begin interpreting these results, we first looked to biochemical factors that may explain the observed ethnic differences in QoL. Other statistically significant ethnic differences in baseline characteristics ‐rates of hypertension, coronary artery disease smoking, and diabetes‐ did not seem to account for the observed differences in QoL. The same is true for systolic blood pressure, levels of log NT‐proBNP, use of alcohol, angiotensin II receptor blocker use, and sex distribution. We must then ask ourselves the following question: what is driving the observed ethnic difference in QoL?

#### 5.1.1. Socio‐economic status and financial burden

In this analysis, areal SEDI—as proxy for socio‐economic status—did not significantly differ among the ethnic groups and as such did not explain any of the variation in QoL seen. Notably, though, >70% of each ethnic group were in the lowest two SEDI (low, low to middle income) categories. This proportion is higher than would be expected in a random sample of the general population, which may reflect an association between low SES and incident HF. However, our data are limited to prevalent HF. Given the low SES of our cohort, the extreme expense of treating HF, and the high (54.8%) out‐of‐pocket expense for healthcare expenditures in Singapore,^19^ “costing you money for medical care” posed the greatest QoL burden to our study cohort.

#### 5.1.2. Historical and cultural factors

Better mean QoL scores in the Chinese at baseline can in part be attributed to the smaller (albeit not significantly different) proportion of patients in NYHA Class III/IV compared to Malays and Indians. The observed ethnic differences in QoL are likely also attributable to ethno‐cultural variance. Notably, the mean MHLFQ total score, 32.9 ± 23.1, in the overall cohort was substantially less severe than those reported in QoL studies focused on Western patient populations, despite the high comorbidity burden.^27^ While this difference might reflect decreased disease severity in Southeast Asian populations, previous analyses have shown that this is not the case.^2^ Key differences lie in the younger age of Asians, with HF occurring almost a decade earlier vs Western cohorts, and the heterogeneity of patients studied. These data also potentially reflect historical and cultural differences in how patients experience, understand, and interpret their disease.

Notably, sexuality, dementia/forgetfulness, and depression are “sensitive” topics which Asians are more hesitant to respond or give neutral responses to (median scores = 0, despite 1070 responses). More than 60% of the patients reported no depression in our study, in contrast to the higher prevalence (though consistent with under‐reporting among Asians patients) as suggested in a meta‐analysis of HF patients.^30^ Another cultural dimension to factor into this analysis is the strong cultural influence of sick Asian (vs Western) patients to have extended family care (in contrast to institutionalism),^31^ although Malay and Indian communities may possess better social networks and experience greater social trust as a result of stronger family and religious ties.^32^ Social and cultural factors, therefore play an important role in shaping people's perceptions and expectations toward health.

#### 5.1.3. Prognostic utility of QoL

Our data suggest that QoL is related to prognosis and that this relationship differs between ethnicities. While the total QoL score was significantly associated with increased mortality hazard in only the Chinese, the physical component was more strongly (twofold) associated with mortality in the overall cohort. This finding is not entirely surprising, since physical symptoms have the simplest and most direct relation to disease course. It is surprising, however, given the fact that QoL does not always align with other risk stratification algorithms. Our findings do suggest, however, that in this cohort improving physical QoL may be correlated with improved prognosis. This is consistent with proven survival benefit of exercise programs in HF. Patients' goal attainment in terms of physical mobility might have implications for rehabilitative medicine for HF patients.^6^

The observation of interethnic differences in the relationship of QoL to prognosis in HF calls for an ethnicity‐specific approach with respect to measures aimed at improving QoL. However, it is important to note that these associations may not reflect a fundamental ethnic difference in HF progression. Any discussion of self‐reported QoL measures is incomplete without calling attention to the multitude of factors that influence QoL which could not be controlled for in our analysis. For instance, at the population‐level, structural and historical ethnic and racial biases play a major role in QoL, which has been well‐documented in the literature on racial health inequities in the United States.^33^ At the individual level, personality traits, such as negative affectivity, developmental experiences, and cultural factors—among others—also influence patients' perceptions of their illness.^11^, ^34^ In addition to these unmeasured biases, our study may be limited by the smaller numbers of Malays and Indians in our cohort.

The study is limited in that global well‐being was not measured with the use of a generic QoL instrument. Additionally, no individual patient socioeconomic variables (eg, household income, education) were available so areal SEDI had to be used as proxy. Despite these minor limitations, a diverse patient population, comprising both inpatients and outpatients, and the availability of comprehensive clinical information, including NT‐proBNP for adjustment and the use of contemporary pharmacotherapy, enhance the generalizability of this population‐based study.

## 6 CONCLUSIONS

In this relatively young cohort of HF patients with high multi‐morbidity, ethnic differences in QoL were seen between Chinese, Malay, and Indian patients. Ethnicity was an independent determinant of QoL. Poorer physical QoL strongly predicted 1‐year survival in the overall cohort. Healthcare professionals should be mindful of such factors to educate patients and their family members so as to provide patients with coping skills to better manage HF. Finally, the findings have implications for an individualized approach to the management of HF patients of different ethnicities and for HF trials that use patient‐reported outcomes as endpoints. Patient‐centered values and QoL should in essence be integrated in clinical decision‐making.

## CONFLICT OF INTEREST

The authors declare no potential conflict of interest.

## Supporting information


**Table S1** Changes in MLHFQ scores at 6 months, by ethnic groups.

**Table S2** Baseline characteristics, included vs excluded patients for subanalysis at 6 month's follow‐up.
