# Supplementary material for: Ethnic differences in quality of life and its association with survival in patients with heart failure
Source: Clin Cardiol. 2020 Jun 20;43(9):976–85. doi: 10.1002/clc.23394 (PMC7462190; doi:10.1002/clc.23394)
Supplement: Supplementary file 1 — Table S1 Changes in MLHFQ scores at 6 months, by ethnic groups. [file CLC-43-976-s001.docx]

**Supplementary Table 1. Changes in MLHFQ scores at 6 months, by ethnic groups**

|  |  |  |  |  |  |  |  |  |  |  |  |
| --- | --- | --- | --- | --- | --- | --- | --- | --- | --- | --- | --- |
|  | All | |  | Chinese | |  | Malay | |  | Indian | |
|  | n | Mean ± SD |  | n | Mean ± SD |  | n | Mean ± SD |  | n | Mean ± SD |
| Minnesota Living with Heart Failure |  |  |  |  |  |  |  |  |  |  |  |
| Total score |  |  |  |  |  |  |  |  |  |  |  |
| Baseline | 622 | 29.7 ± 22.6 |  | 412 | 25.9 ± 20.7 |  | 151 | 36.3 ± 24.2 |  | 59 | 39.5 ± 24.5 |
| 6 months |  | 18.7 ± 19.2 |  |  | 16.0 ± 18.4 |  |  | 25.2 ± 20.2 |  |  | 21.2 ± 18.9 |
| Change in score (6 months - baseline) |  | -11.0 ± 22.5 |  |  | -9.9 ± 21.3 |  |  | -11.1 ± 25.3 |  |  | -18.3 ± 21.3 |
|  |  |  |  |  |  |  |  |  |  |  |  |
| Physical component score |  |  |  |  |  |  |  |  |  |  |  |
| Baseline | 647 | 13.8 ± 10.9 |  | 425 | 12.3 ± 10.2 |  | 162 | 16.2 ± 11.6 |  | 60 | 18.1 ± 11.5 |
| 6 months |  | 8.5 ± 9.3 |  |  | 7.7 ± 9.2 |  |  | 10.1 ± 9.4 |  |  | 9.7 ± 8.9 |
| Change in score (6 months - baseline) |  | -5.4 ± 11.7 |  |  | -4.6 ± 11.1 |  |  | -6.1 ± 13.3 |  |  | -8.4 ± 11.3 |
|  |  |  |  |  |  |  |  |  |  |  |  |
| Emotional component score |  |  |  |  |  |  |  |  |  |  |  |
| Baseline | 645 | 5.6 ± 5.8 |  | 425 | 4.8 ± 5.5 |  | 160 | 7.0 ± 6.2 |  | 60 | 7.4 ± 6.1 |
| 6 months |  | 3.9 ± 5.4 |  |  | 3.4 ± 5.1 |  |  | 5.5 ± 5.9 |  |  | 3.7 ± 5.1 |
| Change in score (6 months - baseline) |  | -1.6 ± 5.8 |  |  | -1.4 ± 5.4 |  |  | -1.5 ± 6.6 |  |  | -3.7 ± 6.5 |
|  |  |  |  |  |  |  |  |  |  |  |  |
|  |  |  |  |  |  |  |  |  |  |  |  |

**Supplementary Table 2. Baseline characteristics, included vs excluded patients for subanalysis at 6 months followup**

| Pulse Pressure, mmHg | Total | Included | Excluded | P value |
| --- | --- | --- | --- | --- |
| n | 1,070 | 649 | 421 |  |
| Age, years | 62.0 ± 12.1 | 61.2 ± 11.7 | 63.2 ± 12.5 | 0.011 |
| Sex, female % | 24.5 | 21.9 | 28.5 | 0.014 |
| Ethnicity, |  |  |  | 0.009 |
| Chinese | 62.3 | 65.8 | 57.0 |  |
| Malay | 26.7 | 25.0 | 29.5 |  |
| Indian | 11.0 | 9.2 | 13.5 |  |
| Clinical characteristics |  |  |  |  |
| Body mass index, kg/m^2^ | 26.3 ± 5.5 | 26.2 ± 5.0 | 26.5 ± 6.2 | 0.477 |
| Systolic blood pressure, mmHg | 125.2 ± 22.4 | 125.3 ± 22.9 | 124.9 ± 21.6 | 0.772 |
| eGFR, mL/min/1.73 m^2^ | 61.2 ± 25.9 | 62.1 ± 25.7 | 59.8 ± 26.1 | 0.164 |
| Biomarkers |  |  |  |  |
| Log NT-proBNP | 7.5 ± 1.4 | 7.5 ± 1.3 | 7.6 ± 1.5 | 0.062 |
| Comorbidities |  |  |  |  |
| NYHA |  |  |  | 0.007 |
| Class I/II | 83.6 | 86.0 | 79.8 |  |
| Class III/IV | 16.4 | 14.0 | 20.2 |  |
| Coronary artery disease, yes % | 53.8 | 53.8 | 53.9 | 0.961 |
| Atrial fibrillation, yes % | 23.1 | 23.6 | 22.3 | 0.599 |
| Hypertension, yes % | 72.2 | 69.4 | 76.6 | 0.011 |
| Prior stroke, yes % | 11.0 | 12.2 | 9.1 | 0.106 |
| Diabetes, yes % | 57.1 | 55.6 | 59.5 | 0.201 |
| Medical therapy |  |  |  |  |
| ACE inhibitor, yes % | 58.8 | 61.0 | 55.3 | 0.066 |
| Angiotensin II receptor blocker, yes % | 12.1 | 12.6 | 11.4 | 0.546 |
| β-blockers, yes % | 87.5 | 89.5 | 84.3 | 0.012 |
| Statins, yes % | 83.4 | 83.7 | 82.9 | 0.741 |
| LVEF, % | 34.7 ± 15.4 | 33.9 ± 14.8 | 35.9 ± 16.2 | 0.050 |
